# COVID-19 and (Im)migrant Carers in Italy: The Production of Carer Precarity

**DOI:** 10.3390/ijerph20126108

**Published:** 2023-06-12

**Authors:** Senyo Dotsey, Audrey Lumley-Sapanski, Maurizio Ambrosini

**Affiliations:** 1 Department of Social and Political Sciences, University of Milan, 20122 Milan, Italy; 2 The Rights Lab, University of Nottingham, Nottingham NG7 2RD, UK

**Keywords:** care workforce, COVID-19, Italy, precarity

## Abstract

This article explores the impact of COVID-19 restrictions on foreign health workers in Italy. Focusing on caregivers in Lombardia, we explore what we call carer precarity, an emergent form of precarity resulting from pandemic restrictions exacerbating existing socio-legal vulnerabilities. The duality of the carer role—complete household and societal reliance in addition to simultaneous socio-legal marginalization—shapes their precarity. Utilizing data from 44 qualitative interviews with migrant care workers in live-in and daycare facilities that were conducted prior to and during the COVID-19 pandemic in Italy, we demonstrate how the migrant populations working in the care sector were particularly adversely affected due to their migratory status and working conditions. Migrants are excluded from or have differential access to a range of benefits or entitlements and are employed in undervalued work. Workers with live-in employment experienced tiered access to benefits plus the spatiality of restrictions, resulting in their near-complete confinement. Drawing on Gardner (2022) and Butler’s (2009) conceptualizations of precarity, we describe the emergence of a new form of pandemic-induced spatial precarity for migrant care workers at the nexus of gendered labor, limited mobility, and the spatiality of and a hierarchy of rights associated with migratory status. The findings have implications for healthcare policy and migration scholarship.

## 1. Introduction

The fight against COVID-19 highlights the importance of migrant workers of all skill levels (low-, medium-, and high-skilled migrants) in alleviating labor shortage problems and contributing to European Union (EU) economies in many vital sectors. In particular, the pandemic drew attention to the role of low-skilled migrants as frontline workers performing “essential work” in Europe, particularly within the health sector [1,2]. This includes care workers who were deemed “critical” and “essential” workers in several European states [3]. Yet this group—migrant care workers—was particularly affected by the forms of the pandemic restrictions. The border closures and lockdown measures adopted during the pandemic significantly impacted care workers’ daily lived experiences. More specifically, the COVID-19 pandemic exacerbated the existing inequalities via restrictions such as further limiting access to public space and the use of public space, limiting spatial mobility and community access, restricting employment mobility, and increasing the psychological burden associated with caregiving responsibilities [3,4]. 

Focusing on migrant care workers in Italy, this article explores the (dual) impacts of the COVID-19 pandemic within the context of state-constructed precarity. While the pandemic affected the employment conditions and well-being of all segments of the population in Europe, migrant populations were some of the hardest-hit groups [5,6]. This was a product of their concentration in the worst-hit sectors—such as the care sector—combined with the multiple forms of vulnerability, risk, exploitation, and precarity produced by their intersectional identities (i.e., visa regimes, migratory status, gender, and ethnicity) [7]. Indeed, this is particularly true in global health and social care systems, which are largely supported by the labor of migrant women. Over 70 percent of frontline health and social care workers worldwide are women, and during the crisis, women were more likely to care for COVID-19 patients in hospitals, care facilities, and private homes [8]. In turn, care workers’ working conditions were more precarious, with increased disease exposure, job instability, and turnover between positions [4]. 

Migrants, and migrant care workers in particular, face state-produced forms of precarity. States influence the protections afforded to workers. Care workers in domestic settings work in “carved-out” spaces in which employment protection and other regulatory laws do not apply. This process of dejuridification—when legal exceptions are made to the application of labor laws—increases the risks of exploitation occurring [9,10]. Further, the state predicates particular benefits and rights on migratory status. This “tiering of entitlement” that flows from socio-legal status limits the options available to migrants who are seeking to meet their basic needs [11]. This includes entitlements to social benefits, welfare, and healthcare, for instance, which flow from the state and without which migrants experience socio-legal and health precarity.

Drawing on interviews with migrant women employed as care workers in the Italian healthcare sector, this paper explores the impact of the pandemic on the lived experience of migrant carers and in particular on the degradation of migrant carers’ labor rights and employment conditions. We find reductions in access to public space combined with fears of job loss and (intermittent) unemployment-driven social isolation, increased responsibilities, and exploitation. This was particularly egregious for live-in carers because of the specific nature of the live-in care model and the embodied responsibilities carers felt to protect their employers. Live-in carers are mandated to spend most of their time in the domestic spaces of their employers’ homes, often lack demarcated schedules, and have limited private space. Existing labor protections within the industry were undercut by pandemic regulations. 

Building on the extant literature on precarity [12] and specifically spatial precarity [13], we demonstrate the impacts of the pandemic’s secondary effects on the care sector, emphasizing the particular spatiality of the restrictions and their influence on care workers’ precarity. Employing Gardner (2022) and Butler’s (2009) conceptualizations of precarity, we describe the emergence of a new form of pandemic-induced spatial precarity experienced by migrant care workers at the nexus of gendered labor, limited mobility, and the spatiality of and the hierarchy of rights associated with migratory status. Our analysis particularly draws attention to the socio-political realities that shape the configuration of the pandemic regulations, the socio-political realities shaping whose protection and rights were prioritized, and the existing access to protections via entitlements that are again reflected in larger gendered and political realities. This paper thus answers the following research questions: How did the pandemic affect the daily lived precarious experiences of the care workforce? To what extent did the pandemic shape carers’ precarious employment conditions?

This paper is organized into five sections. After this introduction, Section 2 documents the relevant literature, the concept of precarity, and the migrant labor force; it then provides an overview of the dynamics of eldercare and COVID-19 in Italy. Section 3 focuses on the research methodology, while Section 4 presents the analysis of the research findings. Section 5 concludes the paper by discussing the research findings and proffering some practical policy recommendations to improve the inclusion of the low-skilled care workforce into the health and social care system.

## 2. Precarity, Migrant Labor Force, and COVID-19

In the period since 9/11 and particularly since the Arab Spring circa 2013, migrants and migration more broadly have been the target of restrictive border policies globally, which were accompanied by the hardening of the internal and external borders of nation-states [14,15]. This is largely a response to the migration threat narrative in which migrants represent a threat to sovereignty and to the cultural cohesion, safety, and security of native populations [16]. This securitization approach involves the amalgamation of actors and resources designed to restrict territorial and socio-legal access to the rights afforded therein [17]. While skilled labor migrants continue to have privileged access, this restrictionist approach has limited the abilities of low-skilled migrants and humanitarian protection seekers to gain territorial access and has led to a proliferation of legislation restricting migrant access to social goods internally (e.g., healthcare and public education). It has been described as the “coming together of people, institutions, resources, laws, territoriality, and mobility” [14] that selectively targets particular forms of migration, functioning to “materialize difference” and target particular bodies as out of place [14]. 

Migrants are often ineligible for state entitlements, or the entitlements are conditional upon aspects of status, for instance, on the length of residency. A complex legal stratification of migrants in many host nations exists as a result, e.g., asylum-seekers, refugees, so-called economic migrants (EU citizens, migrants with short, seasonal, and long-term residence permits), and irregular migrants with an attendant hierarchy of risk and precarity [18]. As Anderson and Andrejsavic write, in this hierarchy of immigration statuses—in which the most vulnerable are those with temporary statuses and limited entitlements—creates marginalized groups without access to a formal labor market or the protection of the state. There are no common rights of movement, livelihood, or societal membership [19]. 

This shapes the protections or exceptions in place based on migratory status [20]. Consequently, migrants with various statuses have differential access to entitlements and socioeconomic and legal rights including the right to work, social welfare benefits, healthcare, and housing [18]. Status may determine the duration of stay, access to rights to remain, threats of deportation, and family reunification [21]. Restrictions on access to entitlements associated with status means that migrants are more likely to lack a social safety net and have fewer resources to fall back on and fewer forms of protection [18].

This hierarchy is further reinforced by the labor force distribution. Access to employment opportunities is associated with legal status which, in turn, affects the distribution of migrant groups by sector, quality, and employer-employee relations [22,23]. Temporary migrants and irregular migrants, for example, often have fewer employment opportunities and are concentrated in the secondary labor market in sectors including agriculture, domestic work, and construction [10,24]. The work in these sectors typically pays less, may be casualized, and requires “long and irregular working hours, and unfair dismissal” [18]. Further, temporary visas may entail fixed contracts or tie laborers to specific employers [25]. Leaving an employer can mean risking irregularization. Given these conditions, temporary migrants are less likely to organize or self-advocate lest they risk deportation, retribution, or abuse. They are also less likely to be in a sector with labor organization or unions [26]. This means that they frequently do not benefit from unionization, such as through attaining secure positions, wages, and benefits. In this way, the state structures opportunities for migrants, leading to a disproportionate concentration of migrants in low-paying positions with less job security and fewer benefits [18,27,28,29].

This hierarchy of migratory statuses then differentially affects population groups, problematizing non-citizens’ livelihoods by linking their legal status with employment rights. This creates vulnerability and precarity through deregulation, tied visas, recruitment fees, fewer employer benefits, employment location and/or conditions, and the temporalities of work contracts [18]. As Anderson writes, “precarious work…is structurally produced by the interaction of employment and immigration” [27]. Gaps in protection by industry (e.g., for domestic workers) and the selective administration of the rights of non-citizens underscores the state’s role in creating an exploitable labor force [11,30]. Judith Butler calls this condition the “differential allocation of precarity” [12]. She describes precarity as:

“precarity” designates that politically induced condition in which certain populations suffer from failing social and economic networks of support and become differentially exposed to injury, violence, and death. Such populations are at heightened risk of disease, poverty, starvation, displacement, and of exposure to violence without protection. [12].

(p. ii)

Butler’s use of precarity describes the way in which the selective lack of protection or provision of positive rights creates differential exposure to deprivation, exploitation, and the abuse of migrants. In this case, precarity captures the experiences of low-wage migrant workers with insecure legal statuses. The visa regimes and employment opportunities coexist to limit opportunities to benefit from the provision of social welfare while affording insufficient wages or benefits. This creates more precarious “life worlds” [18] for groups of migrants clustered in more flexible, less-renumerated positions with risks of exploitation or abuse.

### 2.1. Domestic and Care Workers

The working conditions of domestic workers fall at the intersection of several of these structural and institutional factors [31,32]. First, because of the location of the work, there are exceptions to the provision of labor laws, and the regulation of the workspace is difficult. The working conditions of domestic workers exist a “carved-out” space in which employment protection and other regulatory laws do not apply [27]. In many cases, this is because domestic workers live in their employer’s homes, and exceptions are made for people who live and work in domestic spaces [33]. Attempts at regulation that have been successful do not apply to live-in care workers or domestic workers [33], making it difficult to reach the majority of this population. The regulation or enforcement of laws in the domestic space are problematized by employer–owners who can prevent inspection visits [33]. Further, the threat of information sharing between migration and labor inspection entities can create fear, i.e., of migratory status, for domestic workers which, in turn, limits their willingness to comply with or provide information to authorities. These realities limit opportunities for state interventions to reduce precarity [34].

In certain geographies, domestic workers also suffer from visa restrictions that limit their labor market mobility. The use of tied visas in places such as the UK attaches workers to single households and provides no pathway to change employers. This fixes their wages and conditions of employment for the duration of the contract. In circumstances in which workers have tied visas or their statuses are dependent upon their employer–sponsor, workers may be afraid to leave their employer, causing them to be exposed to abuse [35]. Even in the absence of tied visas, holders of temporary visa have access to fewer entitlements which can leave migrants who lose their work precarious to homelessness, marginality, or destitution [19]. Workers with fewer choices—including domestic workers or carers with tied visas or unstable legal statuses—are less able to protect themselves from exploitation [34]. Employers leverage their positionality for control [36]. 

The gendering of care work [31,37] further impacts the value associated with domestic or care work and, in turn, the parameters around what is expected. Effectively, cultural norms around gender devalue the work of women; this is particularly true of gendered care work, which has been non-renumerated historically [34]. As Banarjee and Wilks summarized, “paid domestic labor remains economically-undervalued, understood as ‘women’s work’, an extension of women’s ‘natural’ domesticity, a reflection of their ‘love’/‘virtue’, and in turn, ‘low-value’ and ‘unskilled’ labor” [37]. As most domestic workers are women, this contributes to a societal condition under which care work is exempted from regulation. The concentration of migrant and ethnic minorities within this sector facilitates further marginalization and exclusion [36]. Additionally, the families who benefit are not always able to afford contracted salaries, leading to an increased likelihood of informality and abuse [31]. 

The pandemic has further impacted the lives of care workers in both institutions and homes (in both formal and informal conditions in several ways) [8,38,39]. On one hand, the pandemic increased work activities for live-in carers without appropriate remuneration [3]. At the other end of the spectrum, carers also experienced reduced working hours and wages and/or experienced unemployment due to decreased economic activity or the threat of exposure to the virus [8,39]. Some live-in domestic care workers were laid off due to preventative precautionary measures and consequently lost their homes [40]. Additionally, those working in institutional settings, even those with stable contracts, saw their working hours reduced, were laid off, or were in fear of losing their jobs because their wards were closed due to COVID-19 deaths and/or low-to-zero intakes of new patients. 

While migrants played a recognized role in keeping the essential service sectors running during the pandemic [41], their distribution in the front-line industries—namely, care work—exposed migrants disproportionately to the pandemic’s negative economic and health effects [18,27,28,29]. While the domestic care sector is essential to EU economies, over the years, this group of migrants has remained outside of EU labor migration management policy initiatives [36,42]. The lack of regulation and the social expectations surrounding care work exacerbated the precarity experienced by this already vulnerable group. 

### 2.2. Italy and Eldercare

Since the 1980s, many postindustrial countries, including Italy, have seen a significant transition in how eldercare is provided [43,44]. In contrast to previous care regimes in which eldercare was left to institutions or family caregivers, seniors now receive an increasing amount of outside support, often from a female foreign workforce, in their homes [43]. In most cases, scholars point to four interconnected factors driving this shift: the increasing participation of women in non-domestic paid work, the simultaneous aging population, welfare policies, and immigration policies (for further details on this, see [44,45].

The aging demographics in many European countries have increased the demand for long-term care services to provide sufficient and skilled care for senior citizens [46]. By 2050, there will be 129.8 million persons in the EU-27 who are 65 years of age or older, an increase from 90.5 million at the beginning of 2019 [47]. Countries such as Italy and Germany have experienced hyper-aging demographics, with fertility rates falling below replacement levels. 

The aging of the European population has led to a high demand for a healthcare labor force. This shift has been accompanied by a reduction in the “welfare state”, which has been shrinking since the early 1980s due to growing concern for financial sustainability. This has contributed to the decision of EU countries to shift “the burden of care management” to families and individual consumers. In the context of commercialization and the neo-liberal logic of care delivery, governments have introduced cash-for-care schemes that aim to push care work out of hospitals [43]. With the lack of adequate and effective institutional arrangements—for example, nursing homes, hospital beds for rehabilitative and long-term care, and the cultural aversion of older adults and families to care institutions—care for older adults is now conceived as a service to be undertaken at home. Consequently, in many European countries, nearly seven in ten elders receive care at home [45]. 

To meet this need, in “welfare states”, a large proportion of older adults are increasingly cared for in their homes by outside workers [43]. These workers are usually female migrants who serve either as daycare providers or cohabiting family caregivers, as in the so-called “migrant-in-the-family” model [48]. Austria, for example, recruits care workers from neighboring countries, such as Bulgaria, the Czech Republic, Hungary, Romania, or Slovakia, of which about 65,000 are personal assistants working in private homes [3]. 

It is worth noting that there is considerable variation in the employment of the migrant workforce in the social care sectors across Europe. While Northern European countries (including Scandinavia and, to some extent, the Netherlands) have historically mostly provided publicly financed social care, such a provision of care was generally inadequate in Southern Europe or modest in continental European countries such as Germany and Austria. Where public care provision was lacking or consisted of unregulated cash benefits, families directly employed a migrant labor force to work for or even live with a dependent family member. These workers are often employed informally and/or have no work or residence permits. Consequently, they lack social and employment protections. This practice is common in places such as Italy, Spain, Greece, Austria, and Germany. In countries where public care provision has been retrenched or marketized, for example, by outsourcing service provision to private providers, a demand often emerges for migrant workers as cheap employees in care institutions or home care providers. This has occurred, for example, in the UK and Sweden [3].

### 2.3. Italian Model of Care

Like most European countries, Italy’s population is experiencing an aging demographic. According to data from the Italian National Institute of Statistics (ISTAT), 14 million people in Italy are 65 years of age or older [49]. This is expected to strain public health and long-term care spending in the ensuing years and decades. Due to decades of underinvestment in public health care, the nation has experienced labor shortages in various areas of the health sector and difficulties in delivering services, with the pandemic making the situation worse [50]. The country is often associated with a familistic welfare care regime: this is explained by the fact that for about 2.8 million elderly persons in need, family, primarily daughters and wives, are responsible for providing care, with over a million foreign workers, as documented later, replacing or supplementing them as live-in or day carers in the past decades [51]. 

After years of generally underdeveloped in-kind services, modern Italian eldercare is centered on the “migrant-in-the-family” model [45]. Instead of the state producing public services over the past few decades, Italy’s reaction to the care deficit has been the implementation of paid-for care programs [52]. Its most significant policy measure is still a type of “Indennità di Accompagnamento” (i.e., companion payment), which is a sum of money that can be spent entirely at the beneficiary’s discretion [53]. The “Indennità” model is untied; it does not call for a monitoring procedure and does not come with referral, information, or guidance services. Consequently, it frequently makes room for employing caregivers from the “shadow or grey market”. 

Interestingly, as indicated earlier, governmental policies appear to accept, if not actively promote, the existence of immigrants in this labor market. Cash-for-care subsidies have thus afforded families opportunities to outsource their caregiving needs to a migrant paid-care workforce [54]. Moreover, this model allows the Italian state to progressively disengage itself (within a neoliberal logic) from traditional welfare functions [43]. 

In 2018, there were approximately 2 million care and domestic workers, of which 859,000 (41.7 per cent) have regular working conditions (according to INPS data)—75.4 per cent of family caregivers and 67.8 per cent of domestic helpers are foreigners—while an estimated 1.2 million (58.3 per cent) work under irregular conditions or are part of the shadow economy. These workers come primarily from Eastern Europe (42.2 per cent), Italy (28.6 per cent), the Philippines (8.0 per cent), and South America (6.8 per cent) [55].

Given the reliance on immigrant women, healthcare workers have received comparatively benevolent amnesties (“sanatorie”) over the years. Non-EU and undocumented domestic workers and caretakers have benefited from generous quotas and regularization measures, the most recent of which targeted healthcare workers in 2009, 2012, and 2020. Italian families have played a crucial role in advocating for the regularization of unauthorized immigrants [56,57]. As Van Hooren posits, this exceptional position of immigrant care assistants can be explained by the help they provide in maintaining a fictional familistic status quo [31]. However, the regularization processes have not been entirely successful over the years. A case in point is the 2020 regularization: the process was fraught with irregularities, a lack of detailed information about the application process, uncertainty about the application outcomes, bureaucratic logjams, long waiting periods, and low acceptance rates. A total of 220,000 people applied under the scheme, and the review of the applications has not ended yet. Allegedly, labor contracts were sold on the black market for EUR 7000 to provide access to visas [58].

While legal regulations and social security are better in Italy than in many other states, live-in carers’ working conditions fall short compared to other employment areas. Some regular caregivers are enrolled with the social insurance agency (INPS), to which they pay pension contributions, but they are not eligible for sickness benefits or unemployment benefits [29,51]. During the pandemic, the government approved an exceptional unemployment benefit for formally employed domestic and care workers (with one or more work contracts of over 10 h per week) who lost a significant portion of their wages. They were eligible to apply for a compensation of EUR 500 per month for April and May 2020; this indemnity, however, excluded live-in domestic workers [47,59]. Under normal conditions, families pay a maximum of 15 days of sick leave (versus other employers, who pay 66.7% of the total wage for 180 days). 

The area is also less regulated. There is no control mechanism or quality assurance system to enforce limitations on work hours or to ensure that workplace conditions meet contractual expectations [60]; see [51] for further discussion. Prior surveys have demonstrated that the lack of regulation or observation may account for a lack of compliance: individuals in live-in carer roles were working 59 h per week versus the 39 worked by those who lived outside their employer’s homes [61]. This underscores the ways in which migrant groups are constructed as precarious, with limited employment options in the context of a less-regulated industry with access to fewer state-provided benefits [61].

More broadly, there are limitations on the rights to benefits for migrants depending on their status and length of residence. For instance, non-EU migrants were not eligible for minimum income supports—“reddito di cittadinanza”—until they had lived in Italy for ten years (and could prove their residence) [50]. Law 388/2000 similarly restricted access to social benefits (e.g., incapacity pension and invalidity allowance) to foreigners for people with long-term residence, generally of up to five years [62]. These policies shape differences in access to benefits and supports that influence an individual’s access to care, their capacity for work, and during times of incapacitation.

In summary, this eldercare system currently appears to be well-ingrained in Italian society because it has proven convenient for all actors involved: Italian families, for a relatively small sum, receive the fundamental assistance they need (i.e., immigrant workers) in the increasingly difficult task of eldercare; immigrants find a way to enter the Italian labor market, and more importantly, if they work as live-in carers, caregivers often solve their housing question and increase the amount of savings they can send back home; and public services may skimp on the resources during a time of the state’s disinvestment in welfare provisions [45]. 

#### COVID Context and Background

Italy was the first country outside China to identify COVID-19. They instituted one of Europe’s most prolonged lockdown measures, with several phases of intermittent partial and full national lockdowns. These were initiated on 21 February 2020 and continued until the beginning of the vaccination campaigns on 27 December 2020, when the country received 9750 doses of the Pfizer–BioNTech COVID-19 vaccine.

The first cases were identified on January 31, 2020, followed by a cluster of cases in the northern Lombardy region shortly thereafter. Following an initial delay, strict and widespread measures were implemented to stop the spread of the virus. The government initially instituted regional restrictions based on the level of threat given the presence of the virus (i.e., a Red Zone indicated the highest risk and was associated with more restrictions). On March 8, restrictions were placed on 14 northern regions, and two days later, the entire country went into lockdown [63]. 

Under these regulations, individuals were not allowed to move between regions and people entering the country were quarantined. There were bans on movement on highways, roads, and railways, public gatherings were banned—including religious ceremonies and sporting events—and schools, universities, theatres, cinemas, bars, and nightclubs were closed [64]. Thermoscanners were used at disembarkation points on arriving passengers or anyone found in public, and police roadblocks were in place [63]. Exceptions to movement regulations for work or health required a government-issued document [65]. Relevant to migrants, authorities froze document processing—e.g., visa applications—at least until 2021 (from 2020 at some point), as most government offices were closed during this period. 

During this period, health workers played an essential role. The extant literature suggests that the pandemic restrictions were particularly impactful on this workforce. We focus specifically on the experience of care workers in the Lombardy region, which was the epicenter of the disease in Italy and the focus of the initial response for much of the initial period of the pandemic in Europe [63]. This region was the focus of much international attention as the world waited to see whether the virus could/would be contained. The mean age of the population (46.1 years), the population density, and the significant number of older residents in Italy more broadly meant that the threat of the disease was a series concern. As a consequence, after an initially slow response, the restrictions—particularly distancing measures—were acknowledged to have been taken seriously and played a role in reducing disease transmission. We explore the form of precarity induced by the government’s pandemic restrictions in Italy, focusing on why and how the pandemic regulations over the use of public space and the intimacy of carer work influenced the lived experiences of carers [66]. 

## 3. Methodology

The results of this study were based on qualitative, in-depth interviews conducted regarding elderly home care in the Lombardy region, Italy. These interviews are part of a larger exploration of the InnovaCARE project: “Enhancing Social Innovation in Elderly Care: Values, practices and policies”. The data collection for the larger project focused on the “triad of eldercare”—eldercare recipients, care managers, and care workers. The interview sample collected for the larger project included 90 interviews, 14 eldercare recipients, 32 care managers (mainly the daughters of the elderly but with a significant portion of husbands and sons), and 44 migrant care workers performing paid live-in and daycare care tasks (all were women except one interviewee). The migrant care workers were aged between 24 and 67, with a mean age of 48.375 (Std. Dev. = 10.428), and were mainly from Eastern Europe and South America. The interviews were conducted between December 2019 and July 2020. Twenty-two of the interviewees were re-contacted to further explore the impact of the COVID-19 pandemic on their lifeworlds. 

The interviews with 44 migrant care workers serve as the basis for the analysis presented herein. All but 6 of the 44 interviewees have some form of a work contract. They work with patients with multiple health conditions, including Alzheimer’s disease, senile/advanced dementia, stroke, old age, physical disability, constant dialysis, motor neuron problems, and heart disease. Caregiving roles require domestic work–cooking and cleaning—in addition to helping patients with their healthcare needs. The lengths of employment ranged from several months to over a decade. The women were single, married, and/or divorced. Some had immediate family in Italy, while others had left their families in their home countries.

Snowball sampling was employed to identify participants. We employed this method in order to reach more “hidden populations” and populations more broadly, given the sensitivity of the topic. The snowball sampling method relies on insider knowledge to identify participants, and it is in this way that we were able to access the population of domestic and care workers interviewed for this study. 

We are aware of the pitfalls associated with this technique—such as the risk of over-sampling migrants who are well-connected. To reduce distortion, we recruited potential migrants through different gateways, including “job-matching service” centers, help desks, immigrant service centers, and previously established personal contacts. This diversity of gateways reduces the problem associated with this method and subsequently improves the quality of the data gathered [67]. 

While the first part of the interview process was carried out in person, the latter part was conducted online due to COVID-19 restrictions. Interviews were recorded and transcribed with the permission of the participants. These transcriptions were coded using thematic coding. The study employed a thematic analysis to answer questions about how the Italian informal welfare arrangement, which is based on the “migrant-in-the-family model”, has fared, particularly during the COVID-19 pandemic. The analysis of the data gathered aimed not to identify the generalizability of results across a population but to use the range of views held within the sample of respondents to serve as the basis of large-scale qualitative and quantitative analyses. To ensure our informants’ anonymity, the names of the research participants were anonymized.

NVivo software was employed to analyze the study’s findings. We coded dominant themes and then identified connections between the emerging themes identified. The themes were clustered into superordinate groups in which relationships were noted and then explicated. Shared themes are explained and illustrated using quotes from the texts.

A note on terminology and use of “low-skilled” migration. In many EU member states, low-skilled labor immigration channels in particular are used to fill mainly short-term positions [68]. Demand for low-skilled migrant labour is high in the old EU15 member states, Cyprus, and Malta, and lower in some EU8 countries (e.g., in the Czech Republic and Hungary). There are no universally accepted definitions of these terms—low-skilled migrants, semi-skilled migrants, and high-skilled migrants—as member states still conceptualize skill levels differently and do not necessarily base residence permits and legislation on these international classifications. Focusing on formal qualifications often hides the fact that skills and experience, which are obtained through on-the-job training, are needed for less-skilled jobs; third-country nationals in particular often have skills that cannot be easily assessed by the certification system or recognized by employers in their destination country [64]. In addition, the labeling of migrants participating in seasonal programs, for example, as low-skilled or semi-skilled migrants, fails to recognize that many of them may actually be highly educated and have solid work experience. In this context, the concept of high or low skills is purely determined by the needs of the host country’s labor market [68].

## 4. Research Findings and Analysis

Using the precarity lens, this section explores the daily lived experiences and employment conditions of caregivers during lockdown. We first present an analysis of the lived experiences of caregivers within the context of the COVID-19 pandemic restrictions, focusing on how the restrictions impacted the individual lives of caregivers. We then explore how the pandemic influenced the aspects of work, examining reductions in wages, increases in unemployment, and losses of hours. Finally, these investigations are drawn together in an analysis of the pandemic’s influence on the precariousness of care workers in the context of the structural and regulatory environment.

### 4.1. Caregivers Daily-Lived Experiences during the COVID-19 Lockdown

*My work as a caregiver: day and night, round the clock, only two hours in the afternoon every day free. Every day of the week. I have been in this profession for almost 9 years. I have been with this patient for 7 years. For now, I care for a man who has the disease Parkinson’s. I have to help him with everything: eating, washing, bathroom, walking, dispensing medicine, cook, clean the house. Everything, except paying bills […] There is ‘friendship’, it’s not just a working relationship, it’s like a family. Because if you don’t consider it like a family, you can’t, because if it’s okay that you just do a few hours and leave, that’s another thing maybe. But when you’re always there, 24 h a day, and so, if you’re treated well, you consider them as your family, not just as work.*

(Mariya, caregiver, Ukraine, 34 years of age)

*I also worked on Sunday and Saturday without anyone paying me […] but I can’t go out; there are police around and everywhere; you can’t, where do I go? In the middle of the street? I’d better stay here at home, huh!*

(Lamia, caregiver, Morocco, 38 years)

Above, Lamia and Mariya describe the lifeworld of the carer workforce and the further impact of the pandemic. Chiefly, Lamia comments on the ways in which the restrictions confined caregivers to their workplaces and the intimate spaces of their employers. This was particularly problematic for live-in carers as they had limited (if any) access to public spaces or other domestic spaces outside their employers’ homes. While Mariya underscores the positive nature of that connection, their comments demonstrate how the regulations exacerbated their containment. This spatial entrapment also contributed to an increase in the number of work hours. 

Caregivers felt “stuck” by the monotony and lack of regulation over their hours. Prior to COVID-19, carers were permitted a two-hour break. During the COVID-19 pandemic, because they were not permitted to leave their homes or move about publicly, most simply remained in their homes. Symbolically, the loss of their usual two-hour daily break most embodied the pandemic’s impact on their day-to-day existences. For live-in care workers, who already had limited free time and were not infrequently tasked above their capacity, this removed their allotted personal time as well as the ability to escape the workplace. Interviewees living with their employers in their workplaces experienced a loss of personal activities they were allowed to or able to pursue. 

The confinement was policed, as was their use of public space, quite literally by ongoing public surveillance. The following interview excerpts from Laminah and Lucie illustrate the impact the confinement had on them:

*Before I had two hours when I can go out, take a walk, have a coffee, that’s the difference, this whole period, almost three months, that I’ve been at home, stuck. The system of my life is normal, like before, I eat, I fix the house. Like before, shower. These things all normal like before. The difference is those two hours … at this time I’m stuck at home, because where do I go? On the street there is no one. The police are around. That’s the difference, I’m stuck.*

(Interview, Laminah, caregiver, Morocco, 38 years)

*[During lockdown] I just worked. I was always at home as prescribed by the law. My day didn’t change too much compared to before the virus (except) I spent my free two hours resting in my room. On Sundays, I rested. My patient’s daughters came, same as before. Only that I stayed at home, that I didn’t go out because I couldn’t go out. I just stayed in the room and that’s all […].*

(Lucie, caregiver, Ukraine, 50 years)

*I spend most of my time here now with my patient and then return home once in a while to my family on the weekend. It’s a bit difficult, but I can’t abandon my patient. I always try my best to also be there for my family.*

(Luis, caregiver, Ecuador, 49 years)

Here, we observe the impacts of spatial restriction on the care workers’ experiences of the pandemic, which confined the social worlds of the workers and their level of autonomy. 

The pandemic and the measures adopted to contain the spread of the virus contributed to a climate of fear and uncertainty for caregivers. Their workplace responsibilities had additional stress due to the threat of COVID-19 itself to their employers, increasing personal worry. Care workers expressed concerns over the attendant risk of their actions to the lives of the patients they served. They adapted their behavior for the patients they served, occasionally to their own detriment. They discussed the impacts this had on their own well-being, leading them to compartmentalize their own needs or wellness.

*[…] in this quarantine, I couldn’t go out for my two free hours. Being a diuretic, I need to walk a little bit—I need to move a little bit. The only difficulty I had after my work has always been like this. The lockdown has impacted my health whilst trying my best to provide care.*

(Assunta, caregiver, Bolivia, 43 years)

*Eh, I went through the pandemic very badly because my sister had this problem, and I couldn’t think about myself. Okay. I couldn’t, because with my lady I had to be smiling all the time, like this, but I could do all this. I was crying somewhere else and smiling in front of my lady, because the lady was a little bit depressed.*

(Kateryna, caregiver, Ukraine)

*Being a single parent, the lockdown isn’t easy because I must attend to my children and the old lady I cared for. It was a challenging moment for everyone involved. I can’t stay home for several reasons: I work in the shadow economy, have no income without working and, importantly, the woman is like a mother to me, so I can’t abandon her.*

(Miriam, caregiver, Senegal, 48 years)

Care workers had the joint tasks of containing the pandemic, as with the majority of the population, and the more specific role of caring for high-risk populations such as the elderly, disabled, and sick. As front-line caregivers during the ongoing crisis, keeping themselves and their patients safe from COVID-19 infection became the focus of their work. This required personal sacrifices that differed in nature from others with familial relationships to the vulnerable. The level of personal sacrifice was associated with their labor. Their decisions to relegate themselves to their workplaces were made in the interest of keeping their employers safe.

This also meant that there were secondary consequences for laborers. Some articulated that they were cognizant that if their employer suffered COVID-19, they too would lose their jobs. For caregivers who provided live-in care, they would also lose their homes and, frequently, their work permits [8]. The excerpt from Lamia, who espoused a view shared by many respondents, highlights this.

*I decided that if I stay at home it’s better for the lady and me because if I may catch the virus, I get well. But if I get the virus and I come here to the house, I feed the lady, and she catches the virus, and right away she goes to the cemetery, huh? That’s why I said from the beginning that I’m afraid more for the lady than for me. That’s the truth because, at this point, this work of mine is where I also live. You understand how it works.*

(Laminah, caregiver, Morocco, 38 years)

This workforce experienced a second level of containment and socio-spatial isolation: restrictions on cross-border mobility disproportionately affected migrants. While many caregivers felt stuck due to restrictions regarding movement from the household, restrictions on national borders contributed to another level of containment and a further feeling of being trapped or stuck. This created or increased workers’ anxieties about their own families and their inability to travel to rejoin or care for them. The lack of knowledge about the duration of the intervention and the permanence of the workers’ containment within their homes and host country had a dual impact. This spatial restriction placed further stress and psychological tolls on the migrant care workers. This was summarized by Lammiah and Aleida:

*[…] I’ve been having a bad time, stressed about everything. I’m away from my family. I’m stuck here because I took the plane ticket on March 14 and after that you know my flight was cancelled. I took the round trip ticket for half a day vacation, and I’m stuck here, all the plans have changed, even before the problem I was already stressed because of my work. I don’t have a lady to lift, to put her diaper, yes. Okay, but my job is all stress, because you have to stay all day and all night at home, necessarily, because my job is like that, twenty-four-hour caregiver. Before the problem, I was already stressed, and I wanted to take a breather. I got the ticket, and everything and I cancelled everything. I’m stuck here, and I’m stuck even more at home and I’ve been through what I’ve been through. Let’s hope well ahead that we don’t go back to quarantine because it’s worse; actually, let’s hope well.*

(Laminah, caregiver, Morocco, 38 years)

*I feel weary and troubled by the situation and can’t return home to see my family. It’s okay to hear through the phone that everything is fine, but this time it’s different because of the pandemic. I must wait until the situation is under control and borders are opened. And I don’t want to leave my patient now that she needs me most—we’re like a family, you know. The right time will come.*

(Aleida, caregiver, Bolivia, 60 years)

### 4.2. COVID-19 and Precarious Employment 

The pandemic restrictions and the fear of the disease spreading disrupted regular work hours; for caregivers without access to unemployment benefits, this meant no income. The pandemic increased unemployment, but it also led to irregular hours, fewer hours, or wage reductions. While caregivers already experienced precarious, low-paying, low-quality, insecure, and irregular employment with differential access to social welfare or labor protections (particularly irregular immigrants), the state interventions to protect the population from the spread of the disease disproportionately impacted this group of workers due to their lack of rights or entitlements to the benefits provided to citizens. Alexis and Aileen underscore the impacts of the COVID restrictions on their employment conditions in the following interview excerpts.

*After this situation, I think some things will change regarding labor conditions. I think there will be a lack of jobs—too many people lost their jobs, even I speak for mine, who work as caregivers also, for us it will be a little bit difficult if we lose a job, right? Finding another person to care for will be a little bit difficult […].*

(Aileen, caregiver, Ecuador, 46 years)

*As you know, the lady with whom I work is quite independent. She left me home during the lockdown because she wanted to be tranquil. Let’s say, because I’m not really fixed all day with her, I have other jobs and I have more contact with other people, and so she said, “It’s better if you stay at home so I’m calmer and so are you.” [What impact did this situation have on you?] “Economically, so much.” Now I started again, it was suspended, let’s say, at the end of March, yes, and I started again then in the middle of May. […] She decided it, let’s say, in order to be more serene, because maybe she said, “And you who are always going round and you can take the disease and then bring it to me” […].*

(Alexis, caregiver, El Salvador, 44)

Again, while the state did provide exceptional benefits to workers due to COVID-19 in lump-sum payments, the lack of regular access to benefits, such as a minimum income, limited the ability of migrant workers to support themselves when their employers reduced their hours. As evidenced by Alexis, the dependency of care workers on their employers’ needs (and vulnerabilities) is understandable, but absent other forms of support for periods without work, it contributes to economic precarity. 

Uncertain and insecure (temporary and marginal) work produces subjective positions that negatively impact all other aspects of health and social life. Many people with legal documents work in this sector without contracts [6,69,70]. It is the lack of contracts—the informality of their employment that causes them to be unable to seek remuneration for lost wages, for instance—that makes them precarious. Further, the fact that employers often prefer informal arrangements can cause laborers to choose this route, shaping their recourse to state support. Kateryna discusses this problem in the case of her sister,

*Eh, for me it was not so difficult, but I had another problem. My sister was working in another family where a lot of people were coming in, nurses, even helpers, even the children back and forth. Then my sister was also sick…the older adults had taken them first to the hospital. The other lady and two died of coronavirus. My sister was sick, and for me, it was a little -- no, not a little, a big worry because she was so ill. Yes, it was a very bad situation. My sister has no documents. She also has problems with the language. She’s been in Italy for a year and a half. She has problems and they were also treating her very badly. I was able to keep in touch with her through telephone every moment I could talk and help her psychologically like that. Today is the first time I met with her, because my family members were afraid if we met that they would take something from me, but today I met with her from a distance, because it’s already been a month and a half since she was sick, and she couldn’t even do the swab to check. But according to me she’s fine, I don’t know, if we can do it. Okay. We are looking for another job. For the residence permit, I’m looking everywhere and I can’t find the job yet. I understand if you don’t have the contract you can’t […] regularize.*

(Kateryna, caregiver, Ukraine)

Kateryna highlights the impact the pandemic had in exposing her sister to illness and trauma. Having lost her job, she had lost her income and her pathway to regularization. This left her precarious and yet, because she was also caregiver, she faced restrictions on supporting her and certainly from taking her in. As one caregiver lamented, the caregiving role exposed caregivers to both risk and illness, while employment contracts and government supports (accessible or otherwise) left them unable to protect themselves. 

Additionally, as the above interviewee indicates, at the pandemic’s outset, care workers in institutions and private homes were working without personal protective equipment (PPE) with people who could be positive for COVID-19 [8,69]. At the same time, for various reasons, the family members could be communicating with outside actors, thus putting their lives, the lives of those to whom they provided services, and the broader public at risk. Further, unlike healthcare workers in hospitals and recognized and established care institutions, a substantial proportion—and perhaps the majority—of private home care workers worked in unregulated locations and under irregular conditions. Though they had the right to “urgent and necessary” medical care or support, as Kateryna indicates, many individuals like her sister were unlikely to seek care due to the risk of exposing their migratory status. Undocumented home care workers work in vulnerable situations as the precariousness of legal position is closely associated with precarious employment and other livelihoods.

The interviews excerpted here support this finding, showing that care workers were inclined to keep their employers safe and to follow pandemic restrictions; however, this led to their isolation, with less access to the forms of activities, breaks, or protections from the pandemic they might have otherwise chosen. This lack of choice—particularly to protect themselves through the use of PPE or to exempt themselves from exposure to the virus present in their workplace—demonstrates how their workplace structures and environments combine to influence precarity. By attaching public benefits to migratory status—including state funds or healthcare access—temporary and irregular care workers had fewer reserves to fall back on or forms of support on which to rely. Further, by not regulating the care industry—and in particular, the domestic sphere—the state allows for conditions which are ripe for exploitation. 

## 5. Conclusions

The paper presents an analysis of the impact of COVID-19 on the cojoined lives and work of care workers in Italy. The domestic care sector experienced the effects of spatial confinement, invisibilization from public spaces, reduced wages/hours/employment, and reduced social contact due to public surveillance. They also lost the ability to have physical contact with individuals who were not their employers. While all populations impacted by these restrictions to a degree, the form of the pandemic restrictions impacted care workers as their homes and livelihoods are cojoined. Some (cohabiting) care workers lost their jobs and consequently, their homes. Others experienced the spectralization of work hours, some with never-ending hours and others with periodic inactivity. This led to psychological problems due to increases in workload, physical confinement, a lack of breaks, and isolation with their patients. While the pandemic restrictions were intended to protect the public from COVID-19, their impacts were unequal and, for this population, akin to those in more institutionalized settings where group wellbeing—in this case, the wellbeing of the care recipients and their families—was protected over individual care workers’ labor rights or personal needs. This reality, combined with a lack of access to full formal state protections or entitlements for this population, created spatial precarity for this group.

Returning to the definition of precarity from Butler and how we reconceptualize its use, Butler [12] defines precarity as “that politically induced condition in which certain populations suffer from failing social and economic support networks and become differentially exposed to …” poverty, displacement, etc. In the case of domestic care workers, precarity resulted from the structural inequalities associated with migratory status and the carved-out exceptions to labor regulations in the industry. The form of pandemic-induced spatial protection taken by the state reduced mobility and public access, impacting health, wellness, and socialization. The physical confinement to intimate domestic spaces, which served both as living and working places, were perhaps the most impactful of the regulations for carers. Therefore, the dejuridification or “space of exception” within the home in which labor regulations do not apply produced this gap in protection [71]. This was exacerbated by the tiered access to social welfare support for migrant groups, which was associated with their length of residency and status. The lack of access to public space for carers and the lack of intervention by the state in private spaces shaped a condition of spatial precarity for carers with increased risks of exploitation and abuse.

We might then add to our conceptualization of carer precarity, employing the lens of what Gardner has called “spatial precarity”, “risks adhering to the configurement of place, emplacement and a person’s relative ability to navigate social and physical space” [13]. Our analysis in particular draws attention to the socio-political realities that shape the configuration of the pandemic regulation, the socio-political realities shaping whose protections and rights were prioritized, and the existing access to protections via entitlements which are reflected, again, in larger gendered and political realities. The preexisting “invisibilization” of women carers’ work in the domestic sphere [37] and the more generalized invisibilization of the gendered work of social reproduction contribute to a lack of state protectionary intervention within that space. The COVID-19 spatial restrictions contained carers to that space without requisite protective labor regulations. Carers also made choices within this context to protect their employers by changing their own behaviors which, due to the nature of their employment, further limited their social interactions. In summary, the pandemic created or shaped a new form of carer precarity that was exacerbated by the spatial forms of pandemic restrictions, which differentially targeted these women in in particular, given their livelihoods and migration statuses. This impact highlights the unevenness in the spatial forms of the pandemic restrictions. 

The development of the COVID-19 pandemic has highlighted the role of migrants, including low-skilled migrants, as frontline workers in essential service sectors (e.g., workers in the agriculture sector, clerks, delivery workers, and healthcare workers) in the EU [7]. Recent research and policy papers show that the public has recognized the significant role of migrants during the pandemic in keeping essential service sectors functioning [3,41], yet it remains unclear whether this positive perception will go beyond the global health crisis and lead to the formulation of more favorable immigration policies [72]. Unsurprisingly, current events show that this positive public outlook was short-lived as countries began to lift COVID-19 lockdown measures. Therefore, policies are needed to ensure the well-being and protection of care workers who have become a key component of long-term care policy and practice in Italy and in Europe as a whole. We propose the following policy recommendations to address the governance of care labor force migration, particularly in light of the pandemic crisis.

Opening legal channels of labor migration for healthcare workers: The hyperaging populations in Italy and the EU bring with them increased needs for long-term care. There is enormous demand for health and care workers and a resulting lack of quality facilities and institutions for the care of older adults, and migrants have played a key role in filling this void. In this context, there is a need to consider tailored, gender-responsive immigration schemes. The ex-post regularization of domestic workers has not been effective in reaching the target population. Care worker visa schemes and the adequate regulation of the industry will somewhat reduce the irregular migration that characterizes domestic and care work migration in particular, as well as the precarious legal and socio-economic conditions that come with it. 

Integration of the care workforce into health and migration governance: To reduce the precarity of migrant carers, it is necessary that they receive adequate health care and the provision of rights. Italy and EU member states must focus on integrating the care workforce into their health and migration governance policies. Importantly, the state should fully incorporate the care labor force into the welfare system by formalizing the sector to help reduce or wipe out the informality that characterizes it. Many care workers will eventually require care themselves in the coming decades. This policy initiative is thus crucial in securing their future when income earnings are lower or non-existent, thus somewhat reducing the burden on governments. 

The separation of patient–employer roles: We recommend separating the client (the patient receiving care) and employer roles. As argued elsewhere [73], an “intermediary agency” that hires female care workforce and then assigns them to clients should be included in the social care management and recruitment process, as is somewhat the process in France.

In essence, the complexity and intractability of the care question necessitate a comprehensive, incremental, and radical structural change in the entire current care system to recognize the multifaceted nature of native–migrant care dynamics in policy formulation and discourses in Europe and to summarily craft workable, win-win solutions that go beyond the one-sided “immoral”care economy that is currently in place.

Further research is, however, needed in this context. A comparison study that explores the experiences of carers in other European contexts with similar population demographics would be useful. In particular, focusing on economies with less informality (the United Kingdom) and states with regulated carer recruitment schemes (Canada) might offer insight into how the pandemic restrictions impacted spatial precarity under different migratory regimes. 

## Data Availability

Data is unavailable due to privacy and ethical restrictions.
